# Space science and technologies to advance health-related sustainable development goals

**DOI:** 10.2471/BLT.17.206565

**Published:** 2018-01-01

**Authors:** Ramesh S Krishnamurthy, Jason Hatton

**Affiliations:** a Department of Information, Evidence and Research, Health Systems and Innovation Cluster, World Health Organization, avenue Appia 20, 1211 Geneva 27, Switzerland.; b Directorate of Human Spaceflight and Robotic Exploration Programmes, European Space Agency, Noordwijk, Netherlands.

In 2018, the world will be celebrating the 50th anniversary of the United Nations Conference on the Exploration and Peaceful Uses of Outer Space (1968–2018): UNISPACE+50.^1^ The use of space science and technologies significantly contributes to our daily lives and has transformative power when applied to public health practice.

Society has already benefited from space technologies, such as satellite television, global positioning and navigation systems, advanced weather forecasting using earth observation data, high-speed global telecommunications, environmental observations and numerous by-products such as car airbags. The applied science programmes of the European Space Agency, Japanese Aerospace eXploration Agency, the American National Air and Space Administration and several other space agencies have produced many technologies that can be applied to health. Leveraging space technologies, such as satellite-based imageries and earth observation data may bring substantial benefits to public health practice. 

Human health, animal health and ecosystems constantly interact; satellite-based geospatial information, including earth observations and remote sensing data, can accelerate our understanding of intricate ecological and environmental interactions and their impact on human health. Analysing these interactions can guide public health decision-making efforts. Large-scale temporal pattern recognition, such as the decline of artic ice over a 20-year period, global deforestation rates or global daily surface temperatures cannot be easily accomplished without earth observation satellites. Satellite-based remote sensing data have been used to identify environmental factors for monitoring Rift Valley fever and other infectious diseases.^2^^–^^4^

Space science and technologies have wide applications, for example in managing public health emergencies, forecasting epidemics, facilitating early warning and disaster management plans, as well as monitoring environmental parameters.^5^ The by-products of space-based technologies and innovations can make substantial contributions to injury prevention from road crashes.

In health services delivery, innovative space technologies are now applied in assistive robotic surgeries, predictive diagnosis, compact water filtration systems, injection safety devices and precision medicine. Furthermore, satellite communications-based tele-health, telemedicine services and tele-guided ultrasound systems now connect patients and caregivers in hard-to-reach or resource-constrained settings. In addition, satellite images can assist in delivering vaccines or accessing health-care facilities by rapidly allowing the detection of road features of an image through feature extraction, producing a map of road networks where maps are either not available or inexistent.

In health systems research, space-based research, such as on the International Space Station^6^ can provide unique data on physiologic and biological processes, which may allow potential novel therapeutic approaches to identify diseases. Space science and technology thus contribute to epidemic intelligence, health emergencies and the research agenda on the benefits to public health.

Space-based innovation in the health sector is poised to bring significant health and economic gains through the adoption of product and process innovations at all levels of health services. This will contribute to delivering better health for all people and could reduce the disease burden. These innovations have a clear place in prevention, preparedness, response and recovery – all the important stages of national public health management. Thus far, the use of space science and technology in public health has been sporadic. Earth observation data from orbiting satellites and ground-based sensors are already used by a few countries to make public health decisions, but more countries could use space-based technologies and geospatial information in this way.

To use space science and technologies in advancing the health sustainable development goal, appropriate national-level policies and governance mechanisms are essential. National governments are encouraged to strengthen policy and governance mechanisms for closer collaboration between health ministries, other relevant ministries and space agencies to leverage the benefits of space science and technologies for health gains. Governments should ensure national technical readiness for geospatial information management as well as the use of space-based innovations and integrate the use of geospatial data in health systems strengthening efforts.

## References

[1] Fifty years since the first United Nations Conference on the Exploration and Peaceful Uses of Outer Space (1968 - 2018): UNISPACE+50. Available from: http://www.unoosa.org/oosa/en/ourwork/unispaceplus50/index.html[cited 2017 Dec 4].

[2] Report on the United Nations/World Health Organization/Switzerland Conference on Strengthening Space Cooperation for Global Health. UN General Assembly document A/AC.105/1161. l New York: United Nations; 2017. Available from: http://www.unoosa.org/oosa/en/ourwork/psa/schedule/2017/conference_who_tp5.html[cited 2017 Dec 4].

[3] Third Meeting of the Expert Group on Space and Global Health, held on 2 and 3 February 2017, and initial considerations in preparation towards UNISPACE+50. UN document A/AC.105/C.1/2017/CRP.28. Vienna: United Nations Office for Outer Space; 2017. Available from: http://www.unoosa.org/res/oosadoc/data/documents/2017/aac_105c_12017crp/aac_105c_12017crp_28_0_html/AC105_C1_2017_CRP28E.pdf[cited 2017 Dec 4].

[4] Report on the meeting on the applications of space science and technology for public health organized by the World Health Organization and the Office for Outer Space Affairs. UN document A/AC.105/1099. Vienna: United Nations Office for Outer Space; 2015. Available from: http://css.unoosa.org/oosa/en/oosadoc/data/documents/2016/aac.105/aac.1051099_0.html[cited 2017 Dec 4].

[5] Space for global health: Special report of the Inter-Agency Meeting on Outer Space Activities on the use of space science and technology within the United Nations system for global health. UN General Assembly document A/AC.105/1091. New York: United Nations; 2015. Available from: http://www.un.org/en/ga/search/view_doc.asp?symbol=A/AC.105/1091[cited 2017 Dec 4].

[6] Report on the United Nations Expert Meeting on the International Space Station benefits for health. UN document A/AC.105/1069. Vienna: United Nations Office for Outer Space; 2014. Available from: http://www.unoosa.org/pdf/reports/ac105/AC105_1069E.pdf[cited 2017 Dec 4].

